# The Association Between Neutrophil-to-Lymphocyte Ratio and Histological Tumor Differentiation in Solid Malignancies: A Systematic Review

**DOI:** 10.3390/diagnostics16142291

**Published:** 2026-07-22

**Authors:** Paul Șiancu, Adina Emilia Croitoru, Cosmin Adrian Teodoru, Gabriela Boța, Monica Pătran, Denisa Tănăsescu, Alexandra-Kristine Tonch-Cerbu, Lilioara-Alexandra Oprinca-Muja, George-Călin Oprinca, Călin-Ilie Mohor, Vicențiu-Vasile Vereș, Maria-Emilia Cerghedean-Florea, Ciprian Tănăsescu

**Affiliations:** 1 Oncology Department, Sibiu County Emergency Clinical Hospital, 550245 Sibiu, Romania; pauls.siancu@ulbsibiu.ro (P.Ș.); mpatran@yahoo.com (M.P.); 2 Department of Dentistry and Nursing, Faculty of Medicine, Lucian Blaga University of Sibiu, 550169 Sibiu, Romania; gabriela.bota@ulbsibiu.ro; 3 Oncology Department, Fundeni Clinical Institute, 022238 Bucharest, Romania; adina.croitoru09@yahoo.com; 4 Department of Oncology, Faculty of Medicine, Carol Davila University of Medicine and Pharmacy, 050474 Bucharest, Romania; 5 Surgical Clinical Department, Faculty of Medicine, Lucian Blaga University of Sibiu, 550169 Sibiu, Romaniatanasescuciprian@yahoo.fr (C.T.); 6 Medical Clinical Department, Faculty of Medicine, Lucian Blaga University of Sibiu, 550169 Sibiu, Romania; denisa.tanasescu@ulbsibiu.ro (D.T.); alexandrakristine.tonchcerbu@ulbsibiu.ro (A.-K.T.-C.); 7 Cardiology Department, Sibiu County Emergency Clinical Hospital, 550245 Sibiu, Romania; 8 Preclinical Department, Faculty of Medicine, Lucian Blaga University of Sibiu, 550169 Sibiu, Romania; lilioaraalexandra.muja@ulbsibiu.ro (L.-A.O.-M.); georgecalin.oprinca@ulbsibiu.ro (G.-C.O.); calin.mohor@ulbsibiu.ro (C.-I.M.); vicentiu.veres@ulbsibiu.ro (V.-V.V.); 9 Surgical Department, Sibiu County Emergency Clinical Hospital, 550245 Sibiu, Romania

**Keywords:** neutrophil-to-lymphocyte ratio (NLR), tumor differentiation grade, histological grading, biomarkers, solid tumors, tumor aggressiveness

## Abstract

**Background:** The neutrophil-to-lymphocyte ratio (NLR) has been the focus of extensive research in recent years as an inexpensive inflammatory biomarker with prognostic value in oncology, but its relationship with tumor grade across solid malignancies remains uncertain. **Methods:** This systematic review evaluated original clinical studies reporting the association between peripheral blood NLR and histological tumor grade in solid tumors. PubMed and Web of Science were searched using Boolean strategies, and eligible full-text studies were synthesized qualitatively because of heterogeneity in tumor type, grading system, NLR threshold, and statistical reporting. **Results:** The qualitative synthesis included 13 retrospective primary studies comprising 5394 patients. Eight studies reported statistically significant associations between higher NLR and higher tumor grade or poorer differentiation, most consistently in bladder cancer and in single studies of prostate cancer, soft tissue sarcoma, renal cell carcinoma, pancreatic cancer, breast cancer, and colorectal cancer with synchronous liver metastases. Four studies reported no statistically significant grade-related association, mainly in heterogeneous breast cancer and ovarian cancer cohorts, whereas a small colorectal adenocarcinoma cohort study showed a non-significant positive trend. **Conclusions:** The available evidence suggests a possible relationship between systemic inflammation and tumor grade in selected malignancies, but the evidence remains preliminary, tumor-specific, retrospective, and vulnerable to confounding. NLR should not be interpreted as a surrogate for histological grade.

## 1. Introduction

Cancer represents a significant global public health problem, and the development of effective control strategies, targeted therapies, and accessible biomarkers remains a priority in contemporary medical practice [1].

Inflammation is a fundamental component of tissue defense and repair; however, in oncology it has a dual biological role. Acute inflammatory responses may support antitumor immunity, whereas chronic inflammation can promote tumor initiation, immune escape, angiogenesis, stromal remodeling, invasion, metastatic dissemination, and therapeutic resistance [2,3].

A distinguishing hallmark of solid tumors is the presence of a complex inflammatory tumor microenvironment composed of malignant epithelial or mesenchymal cells, immune cells, stromal cells, extracellular matrix elements, vascular structures, and soluble inflammatory mediators. This microenvironment exerts a substantial influence on tumor progression and the efficacy of therapy. The tumor microenvironment is a dynamic system in which neoplastic cells constantly interact with stromal and immune components, as well as with the extracellular matrix and inflammatory mediators. Therefore, the tumor can remodel this environment by stimulating angiogenesis and inducing immune tolerance. Concurrently, infiltrating immune cells, including neutrophils, monocytes, and lymphocytes, actively participate in modulating tumor growth and progression [3,4,5].

Neutrophils, which represent the predominant circulating leukocyte population, are functionally heterogeneous. In the context of oncological diseases, they may promote angiogenic signaling, extracellular matrix remodeling, immunosuppressive networks, and neutrophil extracellular trap formation, while also retaining context-dependent cytotoxic activity [6,7]. Lymphocytes are similarly heterogeneous. Cytotoxic CD8+ T-cell infiltration is generally associated with an effective antitumor immune response, whereas regulatory T cells may suppress cytotoxic immunity and contribute to immune tolerance. Peripheral lymphopenia may therefore represent a systemic correlate of impaired antitumor immunosurveillance [8,9].

In medical practice, biomarkers provide clinically relevant information for diagnosis, prognosis, prediction of treatment response, and disease monitoring [10,11,12]. The neutrophil-to-lymphocyte ratio (NLR) is one such biomarker. It integrates two opposing components of systemic inflammation: circulating neutrophilia, reflecting innate inflammatory activation, and relative lymphopenia, reflecting impaired adaptive antitumor immunity. NLR is inexpensive, rapid, reproducible from routine complete blood counts, and extensively investigated as a prognostic marker in non-hematologic malignancies [9,13,14]. Nonetheless, prognostic association and histological grade are distinct endpoints. A marker associated with recurrence or survival does not necessarily reflect tumor differentiation [15,16]. This conceptual distinction is essential for the present review.

Investigating the association between NLR and tumor grade is of potential clinical relevance. If a reproducible association existed, a preoperative complete blood count could provide an early, cost-effective indicator of aggressive histobiology before definitive surgical pathology, potentially accelerating multidisciplinary risk assessment, staging intensity, and treatment-planning discussions [17,18,19].

Histopathological examination remains the diagnostic reference standard for most solid tumors. Beyond confirming malignancy, histological characterization provides prognostically relevant information through tumor architecture, glandular or tissue-lineage differentiation, nuclear pleomorphism, mitotic activity, necrosis, stromal response, tumor budding, and other tumor-specific grading elements. While tumor grade is a morphologic expression of biological aggressiveness, grading systems are not uniform across organs [20,21,22,23].

The research question of this systematic review was: among patients with solid malignancies, is peripheral blood NLR associated with histological tumor grading? The objective was to synthesize available primary clinical evidence, evaluate the direction and consistency of association across tumor types, and identify methodological barriers that currently limit clinical interpretation.

## 2. Materials and Methods

This article was conducted as a systematic review and follows the conceptual structure of the PRISMA 2020 statement [24]. The protocol was not prospectively registered because the project was initially developed as a narrative review and subsequently converted into a systematic review during revision. This is reported transparently as a methodological limitation. A retrospective registration was subsequently completed in the Open Science Framework [25]. Due to the substantial variation among the eligible studies in terms of tumor type, grading scheme, NLR timing, cutoff derivation, statistical model, and effect reporting, a qualitative synthesis was conducted instead of a meta-analysis.

PubMed and Web of Science were searched between 4 February 2026 and 28 March 2026. The Boolean search strategies used for the revised submission were as follows:

PubMed: (“neutrophil-to-lymphocyte ratio” [Title/Abstract]) AND (“tumor grading in malignant cancer” [Title/Abstract]) OR (“histopathological grade in malignant tumors” [Title/Abstract]) OR (“tumor differentiation in malignant cancer” [Title/Abstract]).

Web of Science: (“neutrophil-to-lymphocyte ratio” AND (“tumor grading in malignant cancer” OR “histopathological grade in malignant tumors” OR “tumor differentiation in malignant cancer”)).

Two authors jointly conducted the literature search and screened the retrieved records at both the title/abstract and full-text stages using the predefined eligibility criteria. Any disagreements or uncertainties regarding study eligibility were resolved through discussion and consensus; when uncertainty remained, a third author independently reassessed the relevant article and determined whether it met the inclusion criteria. Data extraction was performed collaboratively by two authors using a predefined extraction framework rather than as independent duplicate extraction. The completed dataset was subsequently verified by a third author against the source articles, and any discrepancies were resolved by consensus.

Eligible studies were original clinical studies of patients with histologically confirmed solid malignancies that reported peripheral blood NLR and directly evaluated its association with tumor grade, histological grade, Gleason grade group, low- versus high-grade classification, or histological differentiation (Table 1). Studies were excluded if they were reviews, systematic reviews, meta-analyses, editorials, commentaries, letters, conference abstracts, book chapters, animal studies, hematologic malignancy studies, case reports, studies without full text in English, or studies focusing only on prognosis, survival, recurrence, radiological response, or treatment outcome without grade-specific NLR analyses.

**Figure 1:**
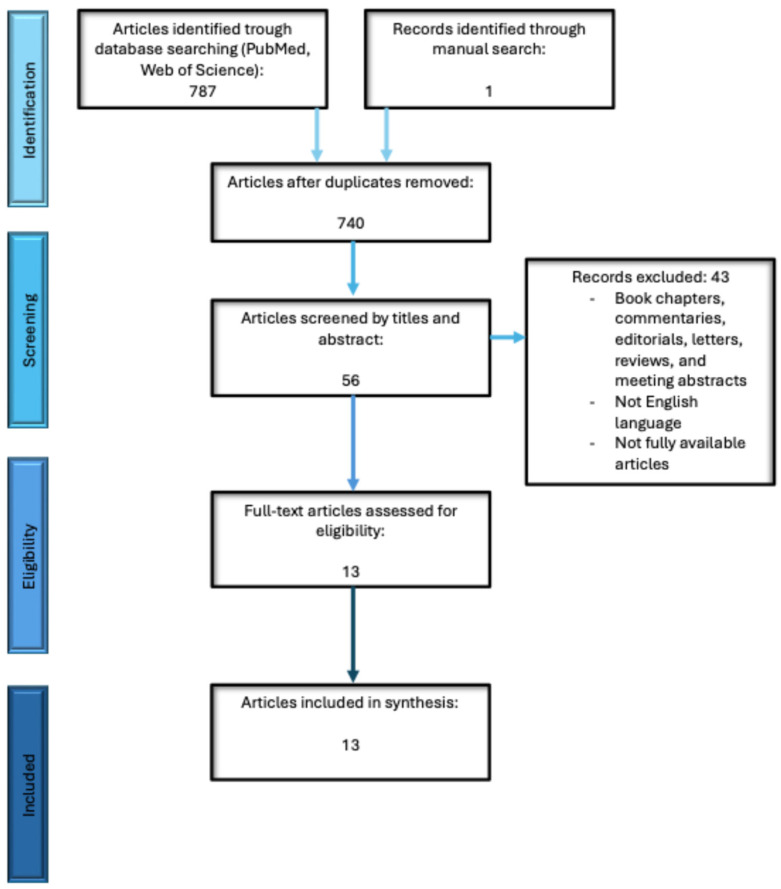
Flowchart illustrating the selection process for the articles included in the review.

Titles and abstracts were screened for relevance, followed by full-text assessment of potentially eligible articles against the prespecified criteria. Only articles available in full text were included, because detailed extraction of grade-specific analyses, NLR timing, cutoffs, and confounder handling was required for this review (Figure 1). For each eligible study, the following items were extracted: first author, publication year, cancer type, sample size, sex distribution when available, disease stage or grade distribution when available, timing of NLR measurement, NLR analysis method, cutoff value, grading system and statistical result for the NLR-grade association (Table 2).

The Newcastle–Ottawa Scale (NOS) was used for observational studies. The NOS evaluates three domains: selection of study groups, comparability of groups, and ascertainment of exposure or outcome. Scores range from 0 to 9. For interpretive purposes, studies scoring 7–9 were considered high quality, 5–6 moderate quality, and 0–4 low quality [26,27]. Individual NOS domain scores are reported as selection/comparability/outcome, together with the total score in Table 3. Across the 13 included studies, NOS scores ranged from 5 to 8, with a median of 7; nine studies were rated as high quality and four as moderate quality.

Because of marked clinical and methodological heterogeneity, no pooled effect estimate was calculated. Synthesis was performed by direction of association and tumor type. Associations were classified as statistically significant positive association, non-significant positive trend, no significant association, or discordant direction. Ambiguous qualitative descriptors were avoided. Where available, exact *p*-values, correlation coefficients, AUC values, or cutoff values were reported rather than qualitative descriptors.

**Table 2:** Study characteristics and NLR measurement details. NLR, neutrophil-to-lymphocyte ratio; M, male; F, female; ROC, receiver operating characteristic. “Not reported” indicates that the item could not be extracted from the source article.

Study	Year	Tumor Type/Setting	Number of Patients (*n*)	Sex (M/F)	NLR Timing	NLR Analysis/Cutoff	Cutoff Method
[28] Nomelini et al.	2019	Ovarian cancer	72	Female-only cohort	Baseline/pretreatment	Categorical, <3 vs. ≥3	Fixed threshold 3.0
[29] Arora et al.	2023	Invasive breast carcinoma	73	Female-only cohort	Baseline/pretreatment	Categorical, <3 vs. ≥3	Fixed threshold 3.0
[30] De La Cruz-Ku et al.	2020	Metastatic triple-negative breast cancer	118	Female-only cohort	At diagnosis/before systemic therapy	Low versus high NLR	Study-defined dichotomy
[31] Mano et al.	2015	Non-muscle-invasive bladder cancer	107	91/16	Preoperative	Median NLR 2.85; cutoff > 2.41	Study-defined cutoff
[32] Jadoon et al.	2023	Invasive breast cancer	2050	Not reported	Baseline/pretreatment	Median NLR 2.14; cutoff 2.5	ROC-derived threshold
[33] Tang et al.	2017	Bladder cancer	302	Not reported	Preoperative	Categorical by NLR 2.5	Study-defined threshold
[34] Oh et al.	2016	Prostate cancer after transrectal ultrasound-guided biopsy	1106	Male-only cohort	Peri-biopsy baseline	NLR higher in high Gleason tumors; cutoff/value 1.83	Comparative analysis
[35] Chan et al.	2018	Soft tissue sarcoma	712	346/366	At diagnosis, before therapy/surgery	Median NLR 4.36 vs. 2.85	Study-defined comparison
[36] Chandrasekaran et al.	2022	Renal cell carcinoma	150	111/39	Preoperative	Cutoff 2.55 for high grade	ROC-derived threshold
[37] Nafissi et al.	2025	Breast cancer	114	Not reported	Baseline/pretreatment	Cutoff 2.15	ROC-derived threshold
[38] Ali et al.	2022	Colorectal adenocarcinoma	46	Not reported	Not reported	Mean NLR increased from well to poor differentiation	Comparative analysis
[39] Kawahara et al.	2024	Pancreatic cancer	461	Not reported	Preoperative	Cutoff 3.2	ROC-derived threshold
[40] Kim et al.	2019	Colorectal cancer with synchronous liver metastasis	83	62/21	Preoperative	Cutoff 1.94	ROC-derived threshold

**Table 3:** NOS = Newcastle–Ottawa Scale. Maximum score: 9 points (Selection = 4, Comparability = 2, Outcome/Exposure = 3). Studies were classified as high quality (7–9 points), moderate quality (5–6 points), and low quality (0–4 points).

Study	Selection (Max 4)	Comparability (Max 2)	Outcome/Exposure (Max 3)	Total NOS (Max 9)	Quality
[28] Nomelini et al.	3	1	2	6	Moderate
[29] Arora et al.	3	1	2	6	Moderate
[30] De La Cruz-Ku et al.	3	2	2	7	High
[31] Mano et al.	3	2	2	7	High
[32] Jadoon et al.	3	1	2	6	Moderate
[33] Tang et al.	3	2	2	7	High
[34] Oh et al.	3	2	2	7	High
[35] Chan et al.	4	2	2	8	High
[36] Chandrasekaran et al.	3	2	2	7	High
[37] Nafissi et al.	3	2	2	7	High
[38] Ali et al.	2	1	2	5	Moderate
[39] Kawahara et al.	3	2	2	7	High
[40] Kim et al.	3	2	2	7	High

## 3. Results

### 3.1. Individual Study Findings

Nomelini et al. (2019) evaluated the association between NLR and histological tumor grade in ovarian cancer (*n* = 72) [28]. The findings did not demonstrate a statistically significant correlation between NLR values and the histological grade. Arora et al. (2023) evaluated the association between NLR and tumor grade in breast cancer (*n* = 73) [29]. No statistically significant relationship was found between NLR and tumor grade. De La Cruz-Ku et al. (2020) evaluated the association between NLR and tumor grade in breast cancer (*n* = 118) [30]. Patients with lower NLR values exhibited higher histological grades; however, this association was not statistically significant (*p* = 0.374). Mano et al. (2015) investigated this association in patients with bladder cancer (*n* = 107) [31]. Elevated NLR values were associated with higher tumor grade. This relationship was statistically significant (*p* = 0.028). Jadoon et al. evaluated the association in a cohort of 2050 patients with breast cancer [32]. The median NLR was 2.14 and the cutoff value determined by ROC analysis was 2.5. No statistically significant association was found between NLR and tumor grade (*p* = 0.694). Tang et al. investigated this relationship in patients with bladder cancer (*n* = 302) [33]. Patients with high-grade tumors had significantly higher NLR values than those with low-grade tumors median NLR 4.42 vs. 3.42, *p* < 0.001). Oh et al. (2016) investigated this association in prostate cancer in a retrospective study including 1106 patients [34]. Higher NLR values were observed in patients with high Gleason score tumors (≥4 + 3) than in those with low-grade tumors or negative biopsies, supporting an association with more aggressive disease (*p* < 0.001). Chan et al. (2018) investigated this relationship in a cohort of 712 patients with soft tissue sarcoma [35]. Elevated NLR values were significantly associated with high-grade tumors and with metastatic disease at diagnosis (*p* < 0.0001). Chandrasekaran et al. (2022) reported a significant association between elevated NLR values and high tumor grade in renal cell carcinoma in a cohort of 150 patients (*p* < 0.05) [36]. Nafissi et al. (2025) evaluated the role of multiple peripheral inflammatory biomarkers in relation to tumor grade in breast cancer in a cohort of 114 patients [37]. Elevated NLR values were significantly associated with poorer tumor differentiation (*p* = 0.027). Ali et al. (2022) evaluated the association between NLR and tumor grade in 46 patients with colorectal adenocarcinoma [38]. Mean NLR values increased across tumor grades; however, the observed trend did not reach statistical significance (*p* = 0.4). Kawahara et al. (2024) evaluated the association between NLR and tumor grade in patients with pancreatic cancer (*n* = 461) [39]. Elevated NLR values (≥3.2) were significantly associated with poorer histological differentiation (*p* = 0.002). Kim et al. (2019) analyzed this relationship in colorectal cancer with synchronous liver metastases (*n* = 83) [40]. Elevated NLR values were significantly associated with poorly differentiated tumors (*p* = 0.048).

### 3.2. Study Characteristics

The final qualitative synthesis included 13 primary retrospective studies comprising 5394 patients.

The included studies evaluated ovarian cancer [28], invasive breast carcinoma [29,32], metastatic triple-negative breast cancer [30], non-muscle-invasive bladder cancer [31], prostate cancer [34], soft tissue sarcoma [35], renal cell carcinoma [36], colorectal adenocarcinoma [38], pancreatic cancer [39], and colorectal cancer with synchronous liver metastases [40]. All studies used peripheral blood NLR, but NLR timing, cutoff derivation, and grade definitions varied substantially (Table 2).

According to the Newcastle–Ottawa Scale, nine of the 13 included studies were classified as high quality, whereas four were rated as moderate quality. No studies were classified as low quality. NOS scores ranged from 5 to 8, with a median of 7 (Table 3).

### 3.3. Direction of Association Between NLR and Tumor Grade

Eight of the 13 included studies demonstrated statistically significant associations between elevated NLR and higher tumor grade or poorer histological differentiation [31,33,34,35,36,37,39,40]. The most consistent evidence was observed for bladder cancer, with both available studies reporting statistically significant positive associations [31,33]. Single studies in prostate cancer, soft tissue sarcoma, renal cell carcinoma, pancreatic cancer, breast cancer, and colorectal cancer with synchronous liver metastases also reported statistically significant positive associations [34,35,36,37,39,40] (Table 4).

The available evidence in breast cancer was heterogeneous. Across the four included studies, one demonstrated a statistically significant association between elevated NLR and poorer histological differentiation [37], two found no significant association [29,32], and one study of metastatic triple-negative breast cancer reported a non-significant inverse trend, with higher tumor grade observed in the lower-NLR group [30]. These findings do not support a consistent association between NLR and tumor grade in breast cancer.

The available evidence for colorectal cancer was similarly inconsistent. In a small cohort of patients with colorectal adenocarcinoma, mean NLR values increased with higher tumor grades; however, the observed trend did not reach statistical significance (*p* = 0.4) [38]. In contrast, a study of patients with colorectal cancer and synchronous liver metastases reported a statistically significant association between elevated preoperative NLR and poor histological differentiation (*p* = 0.048) [40]. The metastatic setting is relevant because tumor burden, hepatic involvement, systemic inflammation, and perioperative physiological stress may influence NLR independently of tumor differentiation.

Ovarian malignancy evidence was limited to one small study reporting no significant grade-related association [28].

### 3.4. Visual Synthesis

**Figure 2:**
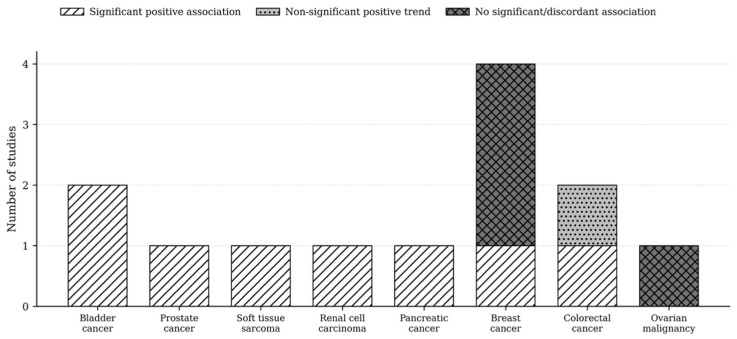
Harvest-style direction-of-effect synthesis of the 13 included primary studies. The figure summarizes the direction and statistical significance of the reported associations according to tumor type and reflects a qualitative synthesis of the available evidence rather than a pooled quantitative effect estimate.

A harvest-style direction-of-effect synthesis was used to summarize tumor-specific patterns without implying statistical pooling (Figure 2) (Table 5).

## 4. Discussion

### 4.1. Principal Findings

This systematic review indicates that an association between elevated NLR and higher tumor grade is biologically plausible in selected solid malignancies, but the overall evidence remains heterogeneous and methodologically fragile. The most consistent evidence was observed in bladder cancer, whereas findings in breast and colorectal cancer were heterogeneous and context-dependent. Because all included studies were retrospective and varied substantially in clinical setting, grading system, NLR measurement, cutoff derivation, and confounder adjustment, NLR cannot currently be considered a reliable surrogate marker for tumor grade.

### 4.2. Tumor-Specific Interpretation

The available evidence was most consistent for bladder cancer. Across studies including patients with non-muscle-invasive and broader bladder cancer cohorts, elevated NLR was consistently associated with high-grade disease [31,33]. This is clinically plausible because high-grade urothelial carcinoma is characterized by architectural disorganization, nuclear pleomorphism, mitotic activity, necrosis, and a greater propensity for invasion and recurrence [41,42]. Nonetheless, bladder cancer studies remain limited in number, and the association may also reflect stage, tumor burden, occult inflammation, or perioperative confounders.

Breast cancer studies were heterogeneous and should be interpreted cautiously. Breast cancer grade is influenced by tubule formation, nuclear pleomorphism, and mitotic count [21]. However, peripheral NLR may be affected by tumor subtype, menopausal status, body mass index, systemic treatment, metastatic burden, and concurrent inflammation [9,32,43]. The discordant findings among invasive breast cancer, metastatic triple-negative breast cancer, and biomarker-focused cohorts suggest that breast cancer should not be treated as a single biological entity when evaluating NLR-grade relationships [29,30,32,37].

The available evidence in colorectal cancer highlights the importance of clinical context. In a small cohort of patients with localized colorectal adenocarcinoma, mean NLR values increased with higher tumor grades, although the observed trend did not reach statistical significance [38]. By contrast, the study including patients with colorectal cancer and synchronous liver metastases demonstrated a statistically significant association between elevated preoperative NLR and poor histological differentiation [40]. In the metastatic setting, hepatic tumor burden may amplify NLR independently of primary tumor grade, limiting causal interpretation [40].

Single studies in prostate cancer, soft tissue sarcoma, renal cell carcinoma, and pancreatic cancer reported positive associations [34,35,36,39]. Each has a distinct grading logic. Gleason grading reflects glandular architectural dedifferentiation [44]; FNCLCC sarcoma grading integrates differentiation, mitotic activity, and necrosis [45]; WHO/ISUP renal grading emphasizes nucleolar prominence and nuclear anaplasia [46]; pancreatic cancer differentiation is embedded in a desmoplastic, immunosuppressive tumor microenvironment [47]. Therefore, a shared NLR signal across these entities may reflect systemic inflammation accompanying aggressive tumor biology rather than a universal grade-specific mechanism.

### 4.3. Relationship to the Broader NLR Literature

The broader oncology literature supports NLR as a prognostic systemic inflammatory marker in many non-hematologic cancers [13,14,17]. However, prognosis and tumor grade are not interchangeable endpoints [48]. A high NLR may reflect tumor burden, occult infection, treatment-related inflammation, cachexia, metastatic disease, host immune status, or comorbid inflammatory conditions rather than intrinsic histological differentiation [9]. The present review therefore extends the current understanding of NLR beyond its established prognostic role by evaluating its potential association with tumor grade, although the available evidence remains inconclusive.

### 4.4. Biological Plausibility

The available evidence suggests a biologically plausible association between elevated NLR and high-grade tumor morphology. Poorly differentiated tumors often display necrosis, hypoxia, proliferative stress, stromal remodeling, genomic instability, and inflammatory cytokine signaling. These processes can recruit neutrophils, expand granulopoiesis, and suppress lymphocyte-mediated antitumor immunity [2,3,4,5,6,7]. Conversely, lymphopenia may mark reduced cytotoxic immune surveillance, whereas neutrophil-dominant inflammation may contribute to angiogenesis, extracellular matrix degradation, immune evasion, and metastatic competence [2,3,5,6,49]. Nonetheless, these mechanisms are not grade-specific. They may also reflect stage, tumor volume, infection, medication exposure, or systemic host factors [9]. This non-specificity is central to the cautious interpretation of NLR.

### 4.5. Clinical Implications

NLR has practical advantages: it is inexpensive, rapid, and available from routine blood counts [50]. However, its clinical applicability remains limited by the absence of a universally accepted pathological cutoff value. Although most studies suggest that NLR values above 3.0 are associated with adverse oncological outcomes, substantial variability exists across the literature, with reported thresholds ranging from approximately 2.5 to 5.0. This lack of standardization hampers direct comparisons across studies and limits the routine clinical implementation of NLR. Consequently, despite its potential as an adjunctive biomarker, NLR should currently be interpreted with caution and integrated with established clinical, pathological, and radiological findings rather than used in isolation [9,51,52].

The study of biomarkers remains a major area of interest in oncology. Although histopathology and clinical staging have traditionally formed the basis for treatment decisions and have provided a practical and effective framework for decades, it has been shown that these factors fail to fully reflect the heterogeneity of the disease and the variability in patients’ clinical course [53,54].

Although modern and emerging biomarkers such as circulating tumor DNA (ctDNA) offer a superior capacity for disease characterization, their use remains limited by cost and availability [55,56]. In this context, the investigation of readily available biomarkers such as NLR is of particular clinical relevance, given their potential for rapid implementation in routine clinical practice. The association between biomarkers and tumor grade may support both diagnostic and therapeutic decision-making. However, the oncological care pathway remains complex and is influenced by multiple factors, including differences between the information provided and the patient’s understanding, as well as the need for close multidisciplinary collaboration among radiologists, pathologists, surgeons, oncologists, and other members of the multidisciplinary team to ensure accurate diagnosis and optimal clinical decision-making. Furthermore, despite ongoing advances in diagnostic techniques, challenges remain that warrant the continued refinement and integration of emerging approaches into routine clinical practice [57,58,59].

### 4.6. Limitations

Several limitations must be emphasized. First, all included studies were retrospective, which introduces selection bias, incomplete confounder control, and variability in clinical data capture. Second, the review included highly heterogeneous tumor types and grading systems, making a pooled meta-analysis inappropriate without harmonized effect measures. Third, NLR thresholds varied substantially across studies, including fixed cutoffs, ROC-derived thresholds, medians, and continuous analyses. Fourth, NLR is non-specific and may be influenced by age, obesity, infection, diabetes, emotional stress, corticosteroid exposure, hematologic disorders, cytotoxic treatment, granulocyte colony-stimulating factor, tumor burden, and metastatic disease [9,13,14]. Fifth, sex distribution, stage distribution, treatment timing, and inflammatory-confounder exclusion were incompletely reported in several source articles and were therefore reported conservatively rather than imputed. Finally, only English-language full-text articles were included, which may introduce language and availability bias.

## 5. Conclusions

The current evidence suggests a possible association between elevated NLR and higher tumor grade in selected solid malignancies, particularly where tumor-specific studies reported consistent positive findings. However, the totality of evidence remains preliminary, retrospective, heterogeneous, and vulnerable to confounding. NLR should not currently be interpreted as a reliable surrogate for histological differentiation or tumor grade. Future studies should be prospective, tumor-specific, and standardized regarding NLR timing, cutoff derivation, inflammatory exclusion criteria, grading definitions, and multivariable adjustment. Until such evidence is available, NLR is best considered a low-cost systemic inflammatory marker of investigational value rather than a clinically actionable marker of tumor grade.

## Figures and Tables

**Table 1:** PICOS framework used for study eligibility.

PICOS Element	Definition Used in This Review
Population	Patients with histologically confirmed solid malignancies
Index	Peripheral blood NLR, analyzed as a continuous variable or as a categorical variable using study-defined thresholds
Comparison	NLR levels compared across tumor-grade or differentiation groups; alternatively, frequency of high-grade tumors compared between low- and high-NLR groups
Outcome	Statistically reported association between NLR and histological tumor grade or differentiation
Study design	Original clinical observational studies or interventional cohorts with extractable baseline NLR-grade data

**Table 4:** Grade-related NLR results and Newcastle–Ottawa Scale assessment. NOS domains are reported as selection/comparability/outcome.

Study	Grade Definition	Direction	Key Statistics	Significant	Adjustment/Confounder Notes	NOS Score
[28] Nomelini et al.	Histological grade	No significant grade-related association	*p* = 0.568	No	Stage-related NLR signal; grade association not statistically significant	3/1/2 = 6 (moderate)
[29] Arora et al.	Histological grade/Ki-67 context	No significant grade-related association	*p* > 0.05	No	Small breast cancer cohort; focused on Ki-67/NLR relationship	3/1/2 = 6 (moderate)
[30] De La Cruz-Ku et al.	Histological grade in metastatic TNBC	Discordant/non-significant direction	*p* = 0.374	No	Primarily early mortality study; grade analysis secondary	3/2/2 = 7 (high)
[31] Mano et al.	Low- vs. high-grade NMIBC	Higher NLR associated with higher grade	R = 0.21, *p* = 0.028	Yes	Hematologic malignancy excluded; retrospective cohort	3/2/2 = 7 (high)
[32] Jadoon et al.	Tumor grade in invasive breast cancer	No significant grade-related association	*p* = 0.694	No	Large retrospective breast cancer cohort; outcome-focused study	3/1/2 = 6 (moderate)
[33] Tang et al.	Low-grade vs. high-grade bladder cancer	High NLR associated with high-grade tumors	*p* < 0.001	Yes	Preoperative NLR; retrospective bladder cancer cohort	3/2/2 = 7 (high)
[34] Oh et al.	Gleason score groups	Higher NLR associated with higher Gleason grade	*p* < 0.001	Yes	Male biopsy cohort; grade represented by Gleason classification	3/2/2 = 7 (high)
[35] Chan et al.	FNCLCC low- vs. high-grade sarcoma	Higher NLR associated with high tumor grade	*p* < 0.0001	Yes	Blood sampled before therapy; infection/hematologic disorders excluded	4/2/2 = 8 (high)
[36] Chandrasekaran et al.	WHO/ISUP low- vs. high-grade RCC	High NLR associated with high-grade tumors	*p* = 0.001; AUC 0.79	Yes	Preoperative cohort; ROC-derived high-grade cutoff	3/2/2 = 7 (high)
[37] Nafissi et al.	High/moderate/poor breast cancer grade	High NLR associated with poorer differentiation	*p* = 0.027; AUC 0.652	Yes	Preoperative inflammatory index analysis; ROC cutoff 2.15	3/2/2 = 7 (high)
[38] Ali et al.	Well/moderate/poor colorectal differentiation	Non-significant positive trend	Mean NLR 4.5 → 5.0 → 6.0; *p* = 0.4	No	Very small cohort; limited statistical precision	2/1/2 = 5 (moderate)
[39] Kawahara et al.	Well vs. poor pancreatic cancer differentiation	High NLR associated with poorer differentiation	*p* = 0.002	Yes	Preoperative surgical cohort; prognostic study with grade analysis	3/2/2 = 7 (high)
[40] Kim et al.	Poorly vs. well/moderately differentiated CRC with synchronous liver metastasis	High NLR associated with poorer differentiation	*p* = 0.048	Yes	Metastatic surgical cohort; NLR cutoff 1.94	3/2/2 = 7 (high)

**Table 5:** Direction-of-effect synthesis by tumor type.

Tumor Type	No. Studies	Significant Positive Association	Non-Significant Positive Trend	No Significant/Discordant Association	Interpretive Note
Bladder cancer	2	2	0	0	Consistent positive direction in both studies
Prostate cancer	1	1	0	0	Grade represented by Gleason classification
Soft tissue sarcoma	1	1	0	0	Single heterogeneous mesenchymal tumor cohort
Renal cell carcinoma	1	1	0	0	Single preoperative surgical cohort
Pancreatic cancer	1	1	0	0	Single surgical cohort
Breast cancer	4	1	0	3	Most heterogeneous tumor-specific evidence
Colorectal cancer	2	1	1	0	Discordance likely influenced by stage and metastatic setting
Ovarian malignancies	1	0	0	1	No association in one small cohort

## Data Availability

No new data were created or analyzed in this study. Data sharing is not applicable to this article.

## Abbreviations

The following abbreviations are used in this manuscript:

AUC	Area under the curve
ctDNA	circulating tumor DNA (deoxyribonucleic acid)
FNCLCC	Federation Nationale des Centres de Lutte Contre le Cancer
NLR	Neutrophil-to-lymphocyte ratio
NOS	Newcastle–Ottawa Scale
PICOS	Population, Exposure/Intervention, Comparison, Outcome, Study design
PRISMA	Preferred Reporting Items for Systematic Reviews and Meta-Analyses
RCC	Renal cell carcinoma
ROC	Receiver operating characteristic
TNBC	Triple-negative breast cancer
WHO/ISUP	World Health Organization/International Society of Urological Pathology
